# Impact of the COVID-19 Pandemic on Health Care Utilization in a Large Integrated Health Care System: Retrospective Cohort Study

**DOI:** 10.2196/26558

**Published:** 2021-04-29

**Authors:** Stanley Xu, Sungching Glenn, Lina Sy, Lei Qian, Vennis Hong, Denison S Ryan, Steven Jacobsen

**Affiliations:** 1 Department of Research & Evaluation Kaiser Permanente Southern California Pasadena, CA United States

**Keywords:** cohort, COVID-19, difference-in-difference analysis, health care utilization, health care worker, impact, knowledge, pandemic, policy, retrospective, telehealth, telemedicine, usage, utilization

## Abstract

**Background:**

The COVID-19 pandemic has caused an abrupt reduction in the use of in-person health care, accompanied by a corresponding surge in the use of telehealth services. However, the extent and nature of changes in health care utilization during the pandemic may differ by care setting. Knowledge of the impact of the pandemic on health care utilization is important to health care organizations and policy makers.

**Objective:**

The aims of this study are (1) to evaluate changes in in-person health care utilization and telehealth visits during the COVID-19 pandemic and (2) to assess the difference in changes in health care utilization between the pandemic year 2020 and the prepandemic year 2019.

**Methods:**

We retrospectively assembled a cohort consisting of members of a large integrated health care organization, who were enrolled between January 6 and November 2, 2019 (prepandemic year), and between January 5 and October 31, 2020 (pandemic year). The rates of visits were calculated weekly for four settings: inpatient, emergency department (ED), outpatient, and telehealth. Using Poisson models, we assessed the impact of the pandemic on health care utilization during the early days of the pandemic and conducted difference-in-deference (DID) analyses to measure the changes in health care utilization, adjusting for the trend of health care utilization in the prepandemic year.

**Results:**

In the early days of the pandemic, we observed significant reductions in inpatient, ED, and outpatient utilization (by 30.2%, 37.0%, and 80.9%, respectively). By contrast, there was a 4-fold increase in telehealth visits between weeks 8 (February 23) and 12 (March 22) in 2020. DID analyses revealed that after adjusting for prepandemic secular trends, the reductions in inpatient, ED, and outpatient visit rates in the early days of the pandemic were 1.6, 8.9, and 367.2 visits per 100 person-years (*P*<.001), respectively, while the increase in telehealth visits was 272.9 visits per 100 person-years (*P*<.001). Further analyses suggested that the increase in telehealth visits offset the reduction in outpatient visits by week 26 (June 28, 2020).

**Conclusions:**

In-person health care utilization decreased drastically during the early period of the pandemic, but there was a corresponding increase in telehealth visits during the same period. By end-June 2020, the combined outpatient and telehealth visits had recovered to prepandemic levels.

## Introduction

The COVID-19 pandemic has caused an abrupt reduction in the use of in-person health care, which has been accompanied by a corresponding surge in the use of telehealth services [1,2]. Health care visits such as inpatient visits, emergency department (ED) visits, and outpatient visits have significantly decreased since the start of the pandemic [3-6]. Two major factors have contributed to these changes. First, patients have chosen not to seek in-person health care owing to the fear of exposure to SARS-CoV-2 [3,7-9]. Second, in the early days of the pandemic, the Centers for Disease Control and Prevention (CDC) and Centers for Medicare and Medicaid Services (CMS) recommended delaying elective care to reduce the risk of SARS-CoV-2 transmission in health care facilities and to reduce the burden on health care systems [10]. Specifically, on March 4, 2020, the governor of California declared a state of emergency after the first official COVID-19 death in the state. On March 19, 2020, a stay-at-home order was enacted in California to slow the spread of SARS-CoV-2.

The CDC also encouraged the use of telehealth services to deliver care [11]. Telehealth is a health care provider’s technology of choice to communicate information regarding the delivery of clinical and nonclinical care services. In addition to providing care for some medical conditions, telehealth has helped protect both providers and patients from the risk of exposure to SARS-CoV-2. It has also helped preserve critical personal protective equipment that was in short supply in the early days of the pandemic.

In response to this, Kaiser Permanente Southern California (KPSC) reported a drastic decline in in-person health care visits, coupled with an immediate increase in telehealth visits. The objectives of this study are to (1) evaluate changes in in-person health care utilization and telehealth visits at one of the largest integrated health care systems in the United States during the COVID-19 pandemic year 2020 and (2) assess the difference in changes in health care utilization between the pandemic year 2020 and the prepandemic year 2019.

## Methods

### Study Population and Study Period

We retrospectively assembled a cohort consisting of members from a large integrated health care system, KPSC. The KPSC serves 4.7 million members at 15 medical centers with at least 50% of its members belonging to racial or ethnic minorities, and 55% living in neighborhoods with a median annual household income of ≤US $75,000 [12]. The study period included the first 43 weeks in the pandemic year (January 5 to October 31, 2020) and the first 43 weeks in the prepandemic year 2019 (January 6 to November 2, 2019). In all analyses, health care utilization was considered for all members of the KPSC enrolled in this study during a given week. Because of data lags in inpatient and ED visits revealed from claims, we only included inpatient and ED visits in the first 35 weeks in the following analyses.

### Data Source and Identification of Visits

We used electronic health record (EHR) data and claims data to identify visits in four settings: inpatient, ED, outpatient, and telehealth. Most of the encounters (approximately 90%) were from EHR data. While EHR data clearly indicated the encounter setting, for claims data, we used place-of-service and hospital revenue codes to determine the encounter setting. Multiple claims were consolidated to resemble a similar visit in the EHR. For example, a consolidated inpatient visit from claims data could include both institutional and professional claims. When a patient was admitted to the ED and then transferred to the hospital, both the ED visit and the hospital visit were considered. For encounters in the outpatient setting, we required a direct interaction between the provider and the patient and a documented diagnosis or procedure code. Encounters for a laboratory test or a procedure only were not included.

For telehealth encounters, telephone appointment visits and video visits were conducted synchronously using real-time telephone or live video-audio interaction, and they were billable and had a diagnosis or procedure code. Thus, telephone appointment visits and video visits were considered telehealth visits in this study. On the other hand, e-visits and message-only encounters were for patient self-triage and for communications without a real-time provider evaluation component. They were not considered telehealth visits in this study. Claims with a telehealth place-of-service code or with the 95 modifier, indicating that the services were delivered through telehealth, were considered telehealth visits in accordance with the CMS billing rules [13].

### Rates of Health Care Utilization During the Pandemic and Prepandemic Years

The rates of visits from these 4 care settings were calculated weekly (Sunday to Saturday) for the prepandemic year and the pandemic year. The numerator was the visit counts of each type, and the denominator was 100 person-years of membership during a given week.

### Statistical Analyses

We first plotted monthly KPSC member enrollment in 2019 and 2020. We examined the demographic characteristics of the cohort, including age, gender, race and ethnicity, and mean Charlson comorbidity index (CCI) of KPSC members in June 2019 and June 2020. CCI scores were calculated only for individuals aged ≥18 years. The visit rates by week during the prepandemic and pandemic years were plotted separately for inpatient, ED, outpatient, and telehealth visits.

In addition to plotting the trends, we used Poisson models to assess the significance of changes in health care utilization after versus before the onset of the pandemic in 2020 relative to changes across the same time periods in 2019, using a difference-in-difference (DID) analysis. To achieve this goal, we selected week 8 (February 23, 2020) as the timepoint before the pandemic because the governor of California declared a state of emergency on March 4, 2020. We also chose week 12 (March 22, 2020) as the timepoint after the start of the pandemic because a stay-at-home order was enacted in California on March 19, 2020. We then selected the 2 corresponding time points during the prepandemic year. In Poisson models, the number of visits was the dependent variable, and an indicator variable for the 2 time points (ie, t=0 for week 8 and t=1 for week 12), an indicator variable for the year (2019 and 2020), and an interaction between the 2 variables were the independent variables. The interaction term was included in the DID analysis to directly assess the significance of the difference in the changes in the visit rates across the 2 years. In these Poisson models, we also included the natural log of person-years as an offset and adjusted for overdispersion of the count data. Because weekly visit data of the entire population were analyzed, individual-level covariates were not included in the analyses.

## Results

### Results Overview

**Figure 1:**
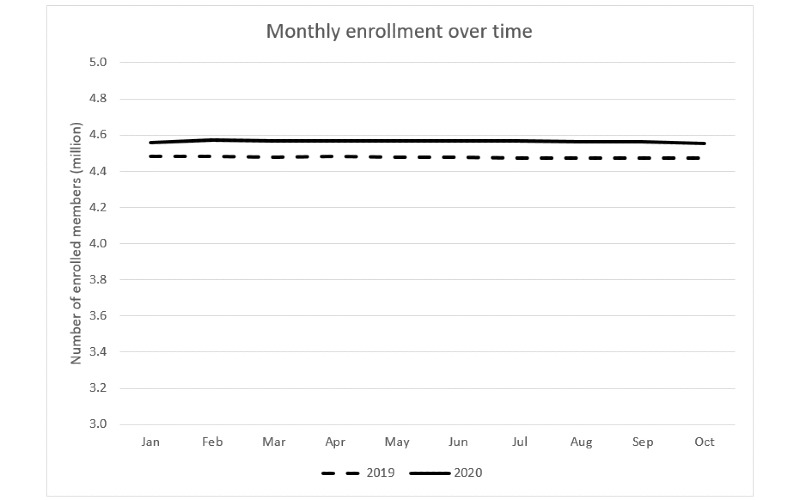
Monthly member enrollment in the Kaiser Permanente Southern California in 2019 and 2020.

Although the member enrollment number in the KPSC slightly decreased from July to October 2020 (4.57 million to 4.55 million), it remained steady during the pandemic year with a range of 4.55-4.57 million, slightly higher than 4.47-4.48 million in 2019 (Figure 1).

**Figure 2:**
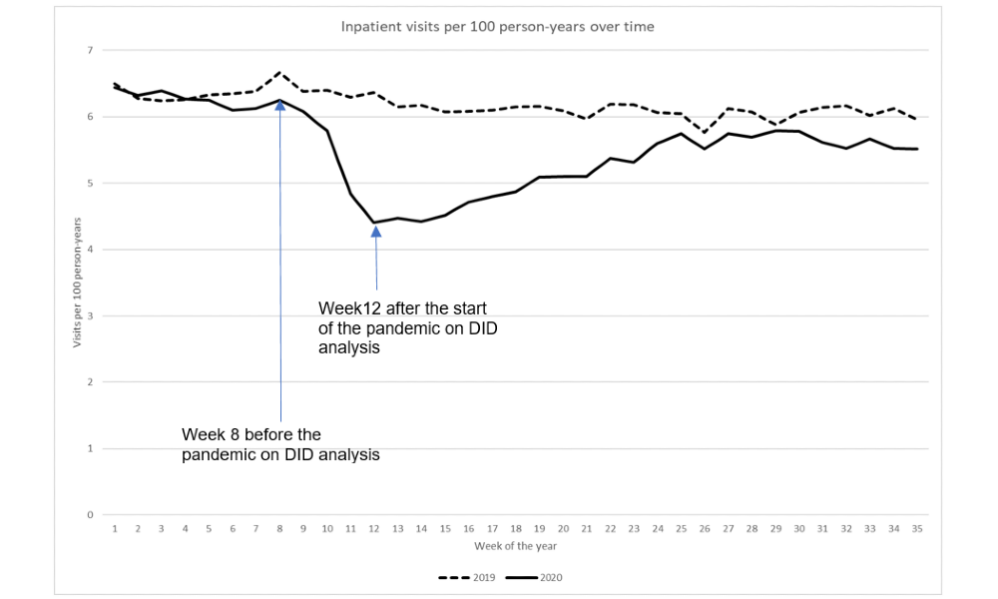
Inpatient visit rate over time. DID: difference in difference.

**Figure 3:**
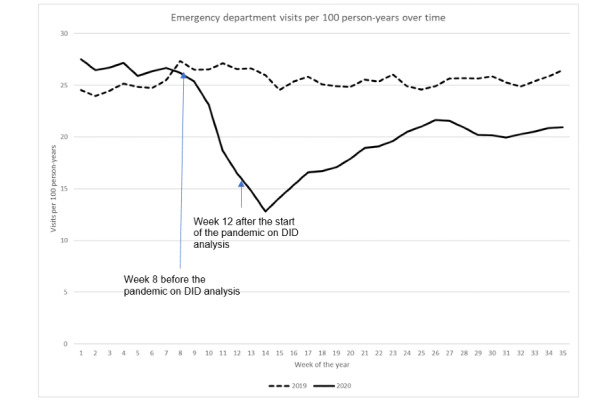
Emergency department visit rate over time. DID: difference in difference.

**Figure 4:**
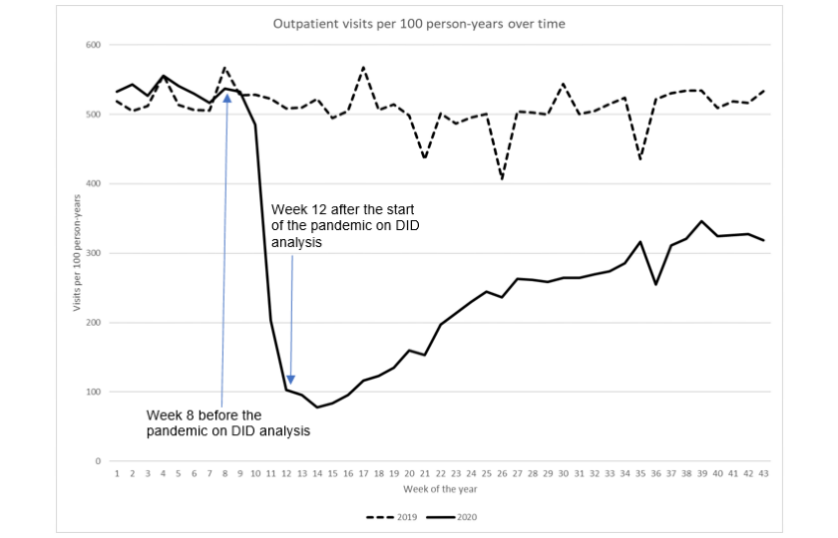
Outpatient visit rate over time. DID: difference in difference.

**Figure 5:**
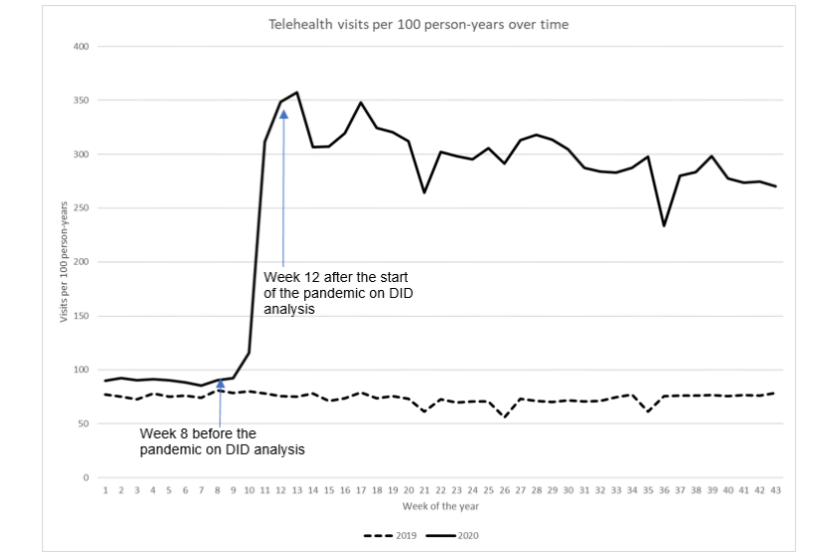
Telehealth visit rate over time. DID: difference in difference.

**Figure 6:**
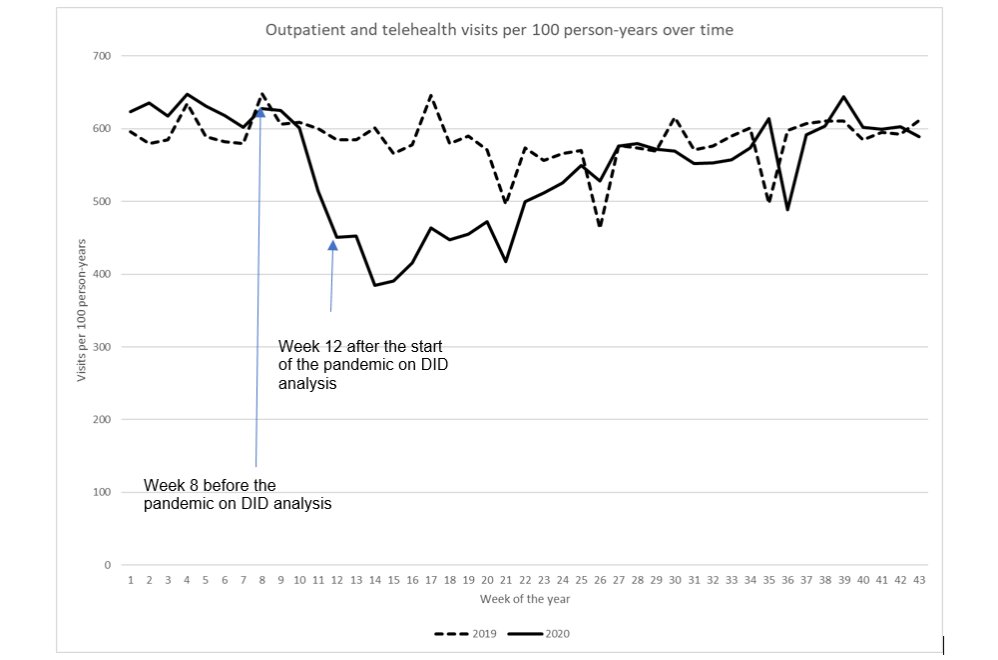
Combined outpatient and telehealth visit rate over time. DID: difference in difference.

Similarly, the characteristics of KPSC members, such as age, gender, race and ethnicity, and mean CCI did not differ between June 2019 and June 2020 (Table 1). The impact of the pandemic on health care utilization in the KPSC was observed after week 8 (February 23) in 2020 (Figures 2-6).

**Table 1:** Demographic characteristics and the Charlson comorbidity index of Kaiser Permanente Southern California members in June 2019 and June 2020.

Demographic characteristics and CCI^a^			June 2019 (n=4,475,819)		June 2020 (n=4,566,641)		
**Age (years), (%)**							
	0-17	20.8		20.9			
	18-44	38.1		39.1			
	45-64	26.2		26.6			
	≥65	14.9		15.4			
Females, (%)		51.5		50.6			
**Race and Ethnicity, (%)**							
	Hispanic	40.9		41.3			
	Non-Hispanic White	31.4		31.0			
	Non-Hispanic Black	7.8		7.8			
	Non-Hispanic Asian or Pacific Islander	11.2		11.3			
	Non-Hispanic Native American or Alaskan	0.2		0.2			
	Non-Hispanic Multiple Races, others, or unknown	8.4		10.4			
Mean CCI (SD)			0.48 (0.96)		0.45 (0.93)		

^a^CCI: Charlson comorbidity index calculated for individuals aged ≥18 years with minimum 1 year of enrollment; n=3,055,756 in 2019 and n=3,115,974 in 2020.

### Inpatient Visits

The inpatient visits per 100 person-years significantly decreased from 6.3 in week 8 (February 23) to 4.4 in week 12 (March 22) during the pandemic year (*P*<.001), thus displaying a 30.2% reduction, while the inpatient visit rate only slightly decreased from 6.7 to 6.4 between the same weeks in 2019 (Figure 2). DID analysis revealed that after adjusting for prepandemic secular trends, the reduction in inpatient visit rates from weeks 8-12 during the pandemic was 1.6 visits per 100 person-years (*P*<.001). After week 12 in the pandemic year, the inpatient visit rate increased until week 30 (July 26) but did not approach prepandemic levels (week 8 in 2020); the inpatient visit rate decreased again after week 30. In contrast, during the prepandemic year, the inpatient visit rate remained at approximately 6 per 100 person-years after week 12.

### ED Visits

ED visits per 100 person-years significantly decreased from 26.2 in week 8 (February 23) to 16.5 in week 12 (March 22) during the pandemic year (*P*<.001), thus displaying a 37.0% reduction, while the ED visit rate slightly decreased from 27.3 in week 8 to 26.6 in week 12 during the prepandemic year (Figure 3). DID analysis revealed that after adjusting for prepandemic secular trends, the reduction in ED visit rates from week 8 to week 12 during the pandemic year was 8.9 visits per 100 person-years (*P*<.001). After week 12 during the pandemic year, the ED visit rate plummeted in week 14 (12.8) and increased to 21.6 in week 27 (July 5), but did not approach prepandemic levels. ED visit rates remained largely unchanged afterwards. In contrast, the average ED visit rate throughout the prepandemic year was 25.5 (range 24.0-27.3) per 100 person-years. The ED visit rates during February 24 to April 6, 2019, were slightly higher than those in the rest of 2019.

### Outpatient Visits

The outpatient visits per 100 person-years drastically decreased from 537.3 in week 8 (February 23) to 102.8 in week 12 (March 22) during the pandemic year (*P*<.001), thus displaying a 80.9% reduction, while the outpatient visit rate slightly decreased from 567.2 to 508.9 for the same period during the prepandemic year (Figure 4). DID analysis revealed that after adjusting for prepandemic secular trends, the reduction in outpatient visit rates from week 8 to week 12 during the pandemic was 367.2 visits per 100 person-years (*P*<.001). After week 12 during the pandemic year, the outpatient visit rate decreased to 77.8 in week 14 and increased to 346.0 in week 39 (September 27), amounting to only 64.4% of the outpatient visit rate in week 8. In contrast, outpatient visit rates fluctuated during the prepandemic year with an average of 502.3 (SD 45.9) per 100 person-years and did not decrease abruptly as it did in 2020.

### Telehealth Visits

In contrast with in-person visits, telehealth visits increased drastically after the onset of the pandemic (Figure 5). While the trend of the telehealth visit rate remained relatively steady during 2019, we observed an approximately 4-fold increase in the telehealth visit rate during the early days of the pandemic year: 90.4 visits per 100 person-years in week 8 (February 23) to 348.3 in week 12 (March 22). Although these rates decreased after week 13, the weekly rate at the end of the study period (October 25) was still almost 3-fold that in week 8. DID analysis revealed that after adjusting for prepandemic secular trends, the increase in telehealth visit rates from week 8 to week 12 was 272.9 visits per 100 person-years (*P*<.001) during the pandemic year.

### Outpatient and Telehealth Visits

To determine whether the increase in telehealth visits offsets the reduction in outpatient visits, we calculated the rate of combined telehealth and outpatient visits (Figure 6). Although not as drastic as the rate of outpatient visits alone, the rate of combined telehealth and outpatient visits decreased from 627.7 visits per 100 person-years to 451.1, thus displaying a 28.1% reduction from week 8 (February 23) to week 12 (March 22) during the pandemic year. After week 12, the rate of combined telehealth and outpatient visits increased, having approached that in week 26 (June 28) in the prepandemic year.

## Discussion

### Principal Findings

In this study, we observed significant reductions in in-person medical visits as the pandemic progressed. The greatest reduction was observed in outpatient visits in the early days of the pandemic (80.9%). Although of lesser magnitude, inpatient and ED visits also decreased by 30.2% and 37.0%, respectively, during the early days of the pandemic. By contrast, we observed an approximately 4-fold increase in telehealth visits in weeks 8-12 in the pandemic year. Further analyses suggest that the increase in telehealth visits did not offset the reduction in outpatient visits during the early days of the pandemic; however, it did compensate for the reduction in outpatient visits by week 26 (June 28). In addition to the CDC recommendation for the use of telehealth services [11], federal and state governments have issued changes in reimbursement policies for these services [14,15]. Even though the pandemic continues to progress with periodic surges in COVID-19 cases and hospitalizations, these policy changes have helped providers deliver health care in telehealth settings.

Our study sheds light on the impact of the pandemic on health care utilization. With approximately 10 months’ data during 2020, this study provides insights into patterns of health care utilization during the pandemic. By using visit rates as our outcomes, we could account for the changes in the underlying population denominator during the pandemic. We observed that KPSC membership generally remained stable during the pandemic, largely owing to the KPSC’s decision to not cancel health coverage for groups or individuals who could not pay for most of the study period. By comparing health care utilization during the pandemic year to that in the prepandemic year through DID analyses, we show that these findings did not result from simply an exacerbation of seasonal effects. Robinson et al [16] recently described the transition to virtual care at the KPSC, but in contrast to our study, they used counts of visits instead of rates as outcomes; hence, they did not adjust for population size. In addition, they included all types of virtual care (including those intended for communication), did not use data from the prepandemic year, and did not conduct a DID analyses. Furthermore, our study included data for 3 additional months.

### Limitations

Some potential limitations in this study must be recognized. First, in addition to the COVID-19 pandemic, other factors such as civil unrest due to racial injustice and the wildfires on the West Coast may have influenced how patients sought health care. We could not differentiate the impact of these factors on health care utilization. Second, these results were derived from a large integrated health care organization that might have been able to change practices quickly, thus potentially not reflecting patterns in other health care systems. Third, while we studied the impact of the pandemic on health care utilization, we did not address the quality of care and population health.

### Conclusions

In conclusion, in-person health care utilization decreased drastically during the early period of the pandemic, but there was a corresponding increase in telehealth visits during the same period. By the end of June 2020, the rate of combined outpatient and telehealth visits reverted to prepandemic levels.

## Abbreviations

CCI: Charlson comorbidity index
CDC: Centers for Disease Control and Prevention
CMS: Centers for Medicare and Medicaid Services
DID: difference in difference
ED: emergency department
EHR: electronic health record
KPSC: Kaiser Permanente Southern California
