# Correction: Impact of the COVID-19 Pandemic on Health Care Utilization in a Large Integrated Health Care System: Retrospective Cohort Study

**DOI:** 10.2196/30101

**Published:** 2021-05-05

**Authors:** Stanley Xu, Sungching Glenn, Lina Sy, Lei Qian, Vennis Hong, Denison S Ryan, Steven Jacobsen

**Affiliations:** 1 Department of Research & Evaluation Kaiser Permanente Southern California Pasadena, CA United States

In “Impact of the COVID-19 Pandemic on Health Care Utilization in a Large Integrated Health Care System: Retrospective Cohort Study” (J Med Internet Res 2021;23(4):e26558) the authors noted two errors.

In the originally published manuscript, the following sentence appeared under the “Data Source and Identification of Visits” section:

They were not considered telehealth consultations in this study.

This sentence has been corrected to:

They were not considered telehealth visits in this study.

In the originally published manuscript, the following sentence was included under the “Principal Findings” section:

We observed that KPSC membership generally remained stable during the pandemic, largely owing to the KPSC’s decision to not cancel health coverage for groups or individuals who could not pay for most of the study period [16].

In the corrected version, the citation to Reference 16 has been removed from this sentence.

The correction will appear in the online version of the paper on the JMIR Publications website on May 5, 2021, together with the publication of this correction notice. Because this was made after submission to PubMed, PubMed Central, and other full-text repositories, the corrected article has also been resubmitted to those repositories.

